# Astemizole, a Second-Generation Histamine H1-Receptor Antagonist, Did Not Attenuate the Aggregation Process of α-Synuclein In Vitro

**DOI:** 10.3390/biomedicines12030611

**Published:** 2024-03-08

**Authors:** Jung Il Choi, Hyunjo Lee, Dong Jun Kim, Eun Suk Park, Kyung Yeon Lee, Hui-Jun Yang

**Affiliations:** 1 Basic-Clinical Convergence Research Institute, University of Ulsan, Ulsan 44033, Republic of Korea; 2 Department of Neurology, Ulsan University Hospital, University of Ulsan College of Medicine, Ulsan 44033, Republic of Korea; 3 Department of Neurosurgery, Wonkwang University Hospital, Wonkwang University School of Medicine, Iksan 54538, Republic of Korea; 4 Department of Pediatrics, Ulsan University Hospital, University of Ulsan College of Medicine, Ulsan 44033, Republic of Korea

**Keywords:** astemizole, synuclein, amyloid, Parkinson’s disease

## Abstract

The antihistamine astemizole has shown disease-modifying effects in several preclinical disease models of Parkinson’s disease (PD). Astemizole also interacts with an anomalous aggregation of Alzheimer’s disease-related amyloid-β (Aβ) peptide and has inhibitory activity on the human prion protein PrP^Sc^. We hypothesized that the proposed preclinical benefits of astemizole on PD can be associated with the attenuation of pathological α-synuclein (α-syn) aggregation. We tested the effects of astemizole on the fibrillation processes of amyloid peptides using thioflavin T aggregation monitoring, Congo red spectral analysis, cell viability study, and transmission electron microscopic imaging. We found that astemizole did not inhibit α-syn aggregation in vitro even at a high molar ratio but inhibited the assembly of Aβ aggregates. Our results suggest that the inhibitory effect of astemizole on amyloid formation is target-protein selective, and the proposed beneficial effects of this compound observed in translational PD models might not be due to its ameliorating effects on α-syn aggregation.

## 1. Introduction

Parkinson’s disease (PD) is the second most common neurodegenerative disease after Alzheimer’s disease (AD). Both neurological disorders carry comparable pathological hallmarks of misfolded amyloid aggregates, such as Lewy bodies associated with α-synuclein (α-syn) in PD or amyloid-β (Aβ) plaques in AD [1,2,3]. α-syn and Aβ are known to have similar prion-like properties, such as seeded fibrillation, adjacent homologous monomers recruitment, cell-to-cell propagation, and resistance to proteases or detergents [2,3,4,5,6,7,8,9]. Two embryonic transplantation studies indicate that α-syn-rich Lewy body pathology may propagate to younger stem-cell grafts from the PD patient-derived host neurons [2,3]. Further supporting studies show that α-syn neuropathology is capable of cell-to-cell transfer in cultured conditions and experimental animal models [7,8]. Peripheral or intracerebral inoculation of pre-formed Aβ aggregates also leads to brain pathology in several translational AD models [4,5,6]. Therefore, α-syn and Aβ peptides are regarded as “prionoid” culprits of both neurodegenerative conditions [1,3,8]. Prionoid proteinopathies such as AD, PD, and tauopathy are tentatively defined by the molecular-level ability to undergo template-directed misfolding, seeded aggregation resulting in a cross β-sheet structure, and intercellular spread to neighboring neurons. However, unlike bona fide human prion disease or mammalian transmissible spongiform encephalopathies, prionoid proteinopathies are not infectious person-to-person [1,9].

Astemizole is a synthetic piperidinyl-benzimidazole derivative initially designed for H1 antihistamine activity and has been used to treat allergic inflammatory symptoms [10,11,12]. Astemizole delays the progression of experimental prion disease by inhibiting the replication of human prion protein PrP^Sc^, and it prolonged survival in an intracerebrally prion-infected mouse model [12,13,14]. Of relevance to prionoid neurodegenerative disorders, astemizole binds and interacts with anomalous aggregates of misfolding-prone Aβ and tau proteins, both of which are associated with AD pathogenesis [15]. Astemizole also modulates PD-related cellular processes, as shown in a microarray study of postmortem brains from patients [16]. Recently, Styczyńska-Soczka and colleagues showed that astemizole improves the survival rate, ameliorates a phenotype of Parkinsonian motor symptoms, and enhances motor performance in a transgenic *Drosophila* model of PD expressing human α-syn in the brain [17].

An increasing number of translational studies suggested that astemizole may have disease-modifying activity in PD, but it is not known if astemizole can block the α-syn fibrillation process as a general amyloid aggregation inhibitor. In addition, astemizole can bind to Aβ aggregates, as explored in previous studies primarily using classical radioligand assays; little attention has been given to the in situ kinetic profiles of astemizole on Aβ fibrillation [15]. In this study, we determined the aggregation-modulating effect of astemizole and its activity on the kinetic profiles of α-syn and Aβ protein fibrillation using in situ real-time fluorescence-based monitoring, Congo red spectral assessment, and transmission electron microscopy.

## 2. Materials and Methods

### 2.1. Materials 

Astemizole, thioflavin T (ThT), and Congo red were obtained from Sigma-Aldrich (St. Louis, MO, USA). Recombinant human α-syn monomer was purchased from Sigma-Aldrich (St. Louis, MO, USA) and Abcam Corporation (Cambridge, MA, USA). The SensoLyte Aβ aggregation kit, which uses recombinant human amyloid-β peptide(1-42) (Aβ42) monomers, was procured from AnaSpec (Fremont, CA, USA). SH-SY5Y wild type neuroblastoma cells were obtained from the Korean Cell Line Bank (KCLB; Seoul, Republic of Korea). The CellTiter 96^®^ non-radioactive cell proliferation assay kit with AQueous One solution reagent was obtained from Promega (Madison, WI, USA). Other reagents and chemicals were procured from Sigma-Aldrich (St. Louis, MO, USA) in analytical grade.

### 2.2. In-Situ ThT Aggregation Assay with Kinetic Analysis

First, 0.5 mg recombinant α-syn monomer was prepared in distilled water and stored at −80 °C. The initial solution of 0.25 mg Aβ42 protein was dissolved in SensoLyte assay buffer and left to hydrate for 5 min. After hydration, the initial solution was centrifuged at 10,000 rpm for 5 min at 4 °C to remove precipitated material. Aβ42 (final concentration: 40 µM) or α-syn (final concentration: 10 µM) were added to a black 96-well flat-bottom plate (Corning, NY, USA) containing various molar ratios (10 µM, 50 µM and 100 µM) of astemizole. Then, 200 µM ThT was added to the plate in a dark room. Fluorescence intensity was measured at 37 °C using a SpectraMax iD3 microplate reader (Molecular Devices, San Jose, CA, USA) and expressed as relative fluorescence units (RFU). Fluorescence readings were obtained every 5 min for Aβ42 studies and every 15 min for α-syn studies at an excitation/emission of 440 nm/484 nm, with pulsed shaking between readings. The fluorescence measurements were performed in at least triplicate.

(1) $$Y=F_{min}+\frac{F_{max}}{1+e^{-\left(\left(t-t_{50}\right)/\tau \right)}}$$

For each molar ratio of astemizole, kinetic analyses of ThT assay data were fitted according to the above sigmoidal Equation (1) [18,19,20], where *Y* represents the detected fluorescence intensity, and *t* and *t*_50_ are the time of aggregation in minutes and the time at which ThT fluorescence reaches 50% maximal intensity, respectively. The apparent rate constant, *k*_app_, is given by 1/*τ* [18]. *F*_min_ is the baseline fluorescence intensity, and *F*_max_ represents the maximum fluorescence at the final saturation phase.

### 2.3. Congo Red Binding Assay and Cell Viablity Study

Amyloid states are characterized by the presence of β-sheet-rich structures that bind to specific dyes including ThT and Congo red. Congo red stock solution was prepared in phosphate buffer (pH 7.4) and filtered with a 0.45 µM filter. Then, 40 µM Aβ42 or 10 µM α-syn solution with or without astemizole (10 µM, 50 µM, and 100 µM) was incubated with the Congo red solution (final concentration: 50 µM) at 25 °C. After 30 min incubation, the absorbance spectra of Congo red binding were spectrophotometrically measured (in arbitrary units) in a transparent 96-well plate at 400–650 nm using a SpectraMax iD3 multi-mode microplate reader (Molecular Devices, San Jose, CA, USA) [20]. Human SH-SY5Y cells (KCLB) were cultivated in minimum essential medium (MEM) supplemented with 10% fetal bovine serum (FBS) for cell cytotoxicity (MTT) assay. Cell cultures were grown in a humidified incubator at 37 °C with 5% CO_2_ (95% air). The cells were seeded at 4 × 10^4^ cells/well density in a 96-well culture plate. After 24 h of incubation, the cell cultures were treated with 10 µM α-syn aggregates along with varying concentrations of astemizole (10 µM, 50 µM, and 100 µM), and separate cell cultures were treated with 10 µM astemizole alone, without α-syn. The cells without any treatment were set as the control group. Cells were incubated for another 24 h, CellTiter 96^®^ AQueous One solution reagent (Promega) was added to the cells followed by a 3 h incubation, according to the manufacturer instructions. For the assessments of cellular toxicity, the astemizole treatment mixtures were collected, and the colorimetric reduction on absorbance at 490 nm was measured using a SpectraMax iD3 microplate reader [21]. Three replicates of each assay were conducted.

### 2.4. Transmission Electron Microscopy (TEM)

Each 10 μL aliquot of untreated and 100 µM astemizole-treated 40 µM Aβ42 and 10 µM α-syn-treated samples were transferred onto the surface of carbon-coated 400-mesh copper TEM grids and negatively stained with 2% uranyl acetate in a dark room. After washing, the dried samples were examined using an FEI Talos L120C electron microscope (Thermo Fisher Scientific, Hillsboro, OR, USA) with a 120 kV acceleration voltage.

### 2.5. Statistical Analysis

Statistical analyses were conducted with Origin version 2022 (OriginLab, Northampton, MA, USA) and SPSS version 24 (IBM, Armonk, NY, USA). The results are shown as the mean ± standard errors of the mean of triplicate data for each experiment. The statistical significance was set at *p* < 0.05. Longitudinal changes in repeated ThT fluorescence measures between groups with different concentrations of astemizole were assessed using a linear mixed-effect model. Other data were analyzed using a one-way analysis of variance with Tukey’s post hoc testing. All graphs were plotted with Origin software, with * indicating *p* < 0.05.

## 3. Results

### 3.1. The Effect of Astemizole on Aβ42 and α-Syn Fibrillation Kinetics in the Real-Time ThT Assay

**Figure 1:**
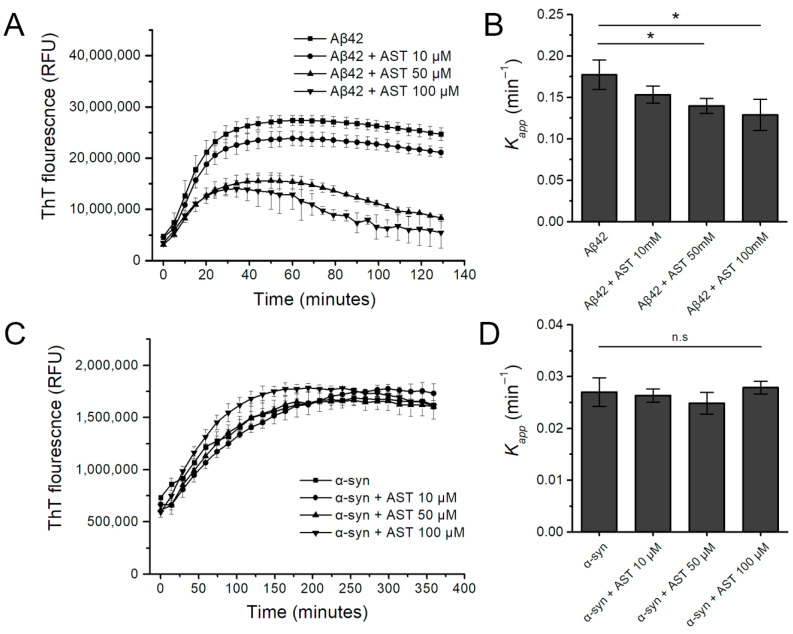
The kinetic effects of astemizole (AST) on the aggregation of amyloid-β(1-42) (Aβ42) or α-synuclein (α-syn) assessed with thioflavin-T (ThT) binding fluorescence in vitro. (**A**,**B**) The aggregation kinetics of 40 μM human Aβ42 monomer in the presence of various concentrations of AST measured by 200 μM ThT-derived fluorescence analysis at 5 min intervals. (**C**,**D**) The aggregation kinetics of 10 μM human α-syn monomer in the presence of various concentrations of astemizole measured by ThT-derived fluorescence analysis at 15 min intervals. Data represent the mean ± standard error from three replicates. * = *p* < 0.05, significantly different compared with Aβ42 monomer (40 μM) or α-syn monomer (10 μM); n.s. = not significant.

The kinetics of Aβ42 and α-syn aggregation in the absence and presence of astemizole are presented in Figure 1. When 40 μM Aβ42 monomer was incubated at 37 °C without astemizole, there was a sigmoidal increase in the ThT fluorescence emission signal. When increasing molar concentrations of astemizole were added, Aβ42 aggregation was reduced compared with the Aβ42 monomer without astemizole. Linear mixed-effect model analysis indicated that the inhibitory activity of astemizole was statistically significant at 50 µM and 100 µM (Figure 1A; *p* = 0.010 and *p* = 0.001, respectively). The apparent rate constant *k*_app_ for Aβ42 amyloid fibril formation decreased by 21.2% at 50 µM astemizole and 26.8% at 100 µM astemizole (Figure 1B; *p* = 0.048 and *p* = 0.045, respectively). By contrast, astemizole did not significantly affect the fluorescence intensity of the amyloid-specific dye ThT when incubated with α-syn (Figure 1C,D).

### 3.2. The Congo Red Binding Assay of Aβ42 and α-Syn Aggregates with or without Astemizole 

**Figure 2:**
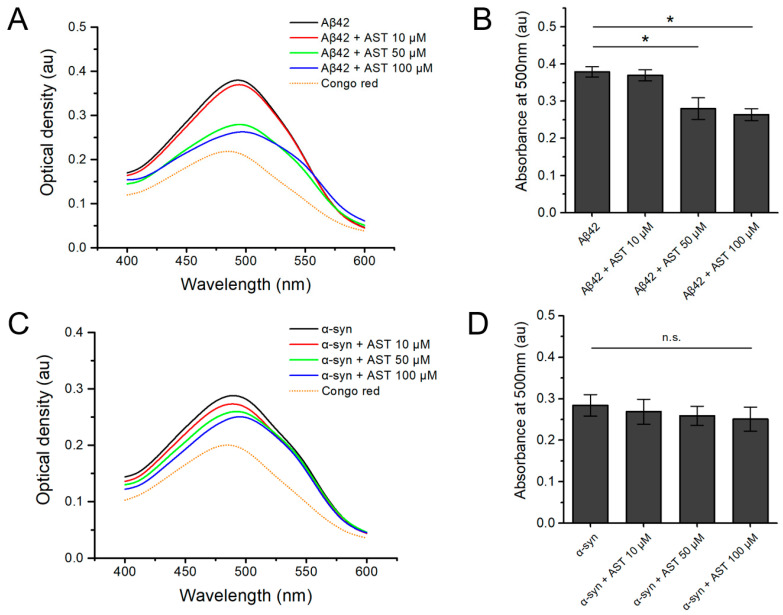
The absorbance spectra of Congo red expressed as arbitrary units (au) in the presence of increasing molar concentrations of astemizole (AST) and human recombinant amyloid-β(1-42) (Aβ42) or α-synuclein (α-syn). The absorbance spectra of Aβ42 or α-syn aggregates formed in the absence (black line) and presence of increasing molar concentrations of AST (colored lines). The absorbance spectra of Congo red alone (dotted yellow line) are also presented. (**A**,**B**) Congo red binding absorbance spectra at 400–600 nm and the binding absorbance of 40 μM human Aβ42 monomer at 500 nm in the presence of various concentrations of astemizole. (**C**,**D**) The absorbance spectra of Congo red in the presence of 10 μM α-syn monomer with or without various concentrations of AST. Data represent the mean ± standard error from three replicates. * = *p* < 0.05, significant compared with Aβ42 (40 μM) or α-syn (10 μM) only; n.s. = not significant.

Next, we assessed the effects of astemizole on protein aggregation using a Congo red binding assay to detect the cross β-sheet structures within human recombinant Aβ42 and α-syn fibrils. Figure 2 shows the spectrophotometrical changes in the relative Congo red absorbance of Aβ42 and α-syn in the presence of different molar ratios of astemizole. The absorption maximum with Congo red alone was 480 nm, and in the presence of Aβ42 or α-syn the maximum shifted to 500 nm. The absorption maximum with Congo red alone was 480 nm, and in the presence of Aβ42 or α-syn, the maximum shifted to 500 nm. The absorption maximum of Aβ42 was significantly altered in the presence of 50 µM astemizole and 100 µM astemizole (Figure 2A,B; *p* = 0.026 and *p* = 0.011, respectively); this result is in agreement with the findings of our ThT kinetic study with Aβ42 monomer.

Conversely, astemizole (10 µM, 50 µM, and 100 µM) did not significantly alter the Congo red absorbance spectra of α-syn, indicating that in vitro fibrillation of α-syn was not affected by astemizole (Figure 1D and Figure 2C; *p* > 0.05).

### 3.3. The Effect of Astemizole on the Aggregation and Cell Viability Assay

**Figure 3:**
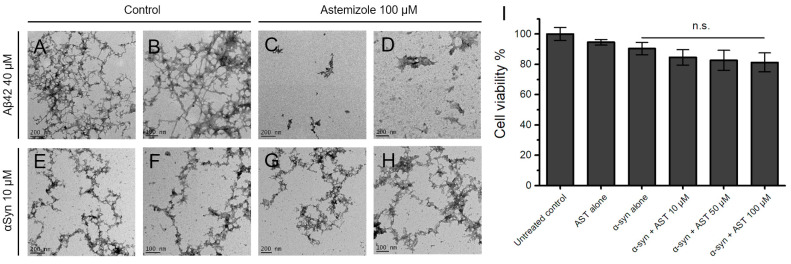
(**A**–**D**) Transmission electron micrographs showing that aggregation of amyloid-β(1-42) (Aβ42) was inhibited in the presence of astemizole. Samples (10 μL) containing 40 μM human Aβ42 protein monomer incubated alone (**A**,**B**) or with 100 μM astemizole (**C**,**D**) were applied to carbon-coated nickel grids, stained with uranyl acetate, and examined at 120 kV voltage. (**E**–**H**) Representative electron micrographs of aggregate morphology with 10 μM α-synuclein (α-syn) alone (**E**,**F**) or in the presence of 100 μM astemizole (**G**,**H**); the images indicate that astemizole did not inhibit α-syn aggregate formation in vitro. The scale bars in each micrograph are 100 nm or 200 nm and are indicated on the image. (**I**) The SH-SY5Y cell viability did not change significantly in cells treated either with α-syn alone or with various concentrations of astemizole (10 µM, 50 µM and 100 µM, *p* > 0.05). n.s. = not significant.

TEM was performed to further characterize the effects of astemizole on morphology of Aβ42 and α-syn aggregates. As shown in Figure 3A–D, negative-stain electron microscopy images indicated that samples of Aβ42 incubated with astemizole formed fewer and smaller aggregates per field than untreated Aβ42. By contrast, it was apparent from the results shown in Figure 3E–H that astemizole failed to inhibit α-syn aggregation; this finding agrees with results from our ThT fluorescence-based monitoring and Congo red binding assay. Furthermore, Figure 3I shows the impact of astemizole on the survival of α-syn aggregate-treated SH-SY5Y cells in comparison with the control group. The viability of human-derived neuro-blastoma cells remained largely unaffected whether exposed to α-syn alone or following treatment with different doses of astemizole (10 µM, 50 µM, and 100 µM, *p* > 0.05).

## 4. Discussion

There is a growing body of preclinical and clinical evidence suggesting that α-syn and Aβ share the prion-like properties that are crucial for the depositing and spreading of amyloids across the cerebral regions [1,2,3]. Braak et al. made early observations of the postmortem brains of PD patients and found temporal patterns of stereotypical involvement in certain brain regions over long periods [2,3]. These neuropathological patterns manifested even before the initial presentation of clinical PD [3,8]. The Braak staging based on these observations has been increasingly accepted as a pathological corroboration for the hypothesis that the initial exogenous insults trigger the condition as knocking over the first vulnerable domino and the subsequent inter-neuronal spreading sequentially throughout the nervous system as more dominoes fall [2,8,9]. In 2008, another key finding that supports the prion-like mechanisms underlying neurodegenerative disorders came from the therapeutic trials of the fetal cell implantation in patients with advanced PD [2,3]. Lewy bodies were discovered in 3–5% of fetal implants a decade or more after the initial transplantation of embryonic tissue. These Lewy bodies reached up to ~30% of graft cells, increasing over time with the survival duration of recipients [3]. Alongside the clinical transplant studies, accumulating experimental data indicated that recipient cell lines and susceptible transgenic mammals can develop progressive synuclein pathologies following the application of recombinant α-syn preformed fibrils (PFF) [7,8,9]. Direct cerebral and peripheral inoculation with α-syn PFF in wild-type rodents and non-transgenic primates can also induce cerebral Lewy body formation [3,7]. In AD, several translational studies have demonstrated that the addition of preformed Aβ aggregates to a monomeric solution speeds up fibril growth, and the surfaces of existing PFF can aid the conversion of monomers into amyloid aggregates [3,5,6]. These in vitro findings support the results from in vivo Aβ seeding studies, demonstrating the acceleration of amyloid formation and deposition following an injection among mammals prone to amyloid deposits with extracellular Aβ [4,5,6,9]. Moreover, a recent study in which intracerebral human Aβ oligomers were injected into wild-type rats observed that social memory impairments were proportional to the decrease in lateral entorhinal cortical volume [4].

However, it is well known that PD and AD are not transmissible naturally between humans in the way that human prion diseases are, even though misfolded α-syn and Aβ have similar properties to the PrP^Sc^ prion protein at a molecular level [1,9]. Therefore, the umbrella term prionoid proteinopathy is used to describe various progressive neurodegenerative disorders that are characterized by the accumulation and propagation of prion-like amyloid proteins within neurons or the brain parenchyma without evidence of person-to-person infectivity.

This prionoid proteinopathy model has important implications for developing therapeutic strategies for PD and AD. To stop the falling dominoes, many types of anti-amyloid molecules have been tested that attempt to target relevant molecular processes, including blocking intercellular propagation, inhibiting aggregation, promoting chaperone interactions, and upregulating lysosomal degradation and autophagy [9,14,22]. However, de novo-designed anti-amyloid compounds have suffered from high costs, unexpected adverse effects, contradictory clinical results, and protracted timelines [22]. Drug repurposing or repositioning, which refers to the use of drugs previously licensed for other indications, is becoming a viable approach for prionoid disorders, and offers faster and cost-effective development combined with largely accessible safety and toxicity profiles [10,12,22]. This approach prompted us to investigate the list of known anti-prion compounds originally targeting bona fide human prion disease, which also have shown therapeutic potential in prionoid neurodegenerative disorders.

Astemizole, a selective histamine H1-receptor antagonist, fits our drug-repurposing criteria; this compound was shown to have anti-prion properties in a high-throughput screen of a library of 1280 US-approved drugs [12,13]. After its initial discovery in 1977 by Janssen Pharmaceutica, astemizole was used for treating allergic symptoms, including rhinitis and urticaria, as a prescription or over-the-counter medication in over 100 countries [10]. In 1999, however, astemizole was voluntarily withdrawn from the United States and European markets after declining sales in the wake of rare cardiac side effects, and the introduction of newer and safer antihistamines [12,23,24]. Generic astemizole is currently sold in about 30 countries [10,12], and cardiac deaths attributed to astemizole are fewer than one case per 100 million doses based on a decade of surveillance data from 17 countries [25].

Astemizole inhibits prion protein replication in cell lines infected with 22L scrapie strain or Rocky Mountain Laboratory (RML) prion strain [12,13,14]. Encouragingly, several recent studies have demonstrated that astemizole has comparable brain permeability [11,15]. The anti-prion effects of astemizole have been tested in a rodent model following intracerebral inoculation with an RML strain, and there was a marginal increase in the survival time of prion-infected mice treated with astemizole [12]. Static radioligand assay studies have shown that astemizole interferes with abnormally aggregated Aβ peptides; however, the kinetics of this Aβ aggregation have not been elucidated. Furthermore, few studies have determined whether astemizole alters α-syn aggregation and fibrillation [15,17]. We hypothesized that the proposed beneficial effect of astemizole seen in preclinical models of PD is associated with inhibition of the amyloid precipitation of α-syn, as has been suggested for other prionoid amyloid proteins, including Aβ [15,16,17,21,26].

We found that the formation of Aβ42 fibrils was attenuated by astemizole, which was in line with a previous study that used a radioligand assay [15], but astemizole did not prevent the amyloid assembly of α-syn, even at a high molar ratio. Our results suggest that the ameliorative effects of astemizole on the formation of amyloids are rather protein-specific, while previously reported small-molecule inhibitors or chaperones can inhibit the formation of a range of amyloid proteins [26,27]. For example, epigallocathecin-3-gallate and several polyphenol molecules inhibit the aggregation of Aβ, α-syn, tau, human islet amyloid polypeptide, and the prion protein Sup35 [26]. Molecular chaperones including intracellular heat-shock proteins have anti-amyloid activities on α-syn, Aβ42, tau, and SOD1 proteins [27,28,29]. The observed target-amyloid selectivity of astemizole is not unprecedented; a previous study showed that astemizole displayed different affinities for Aβ42 and tau aggregates [15], and further studies are needed to determine the reasons for the proposed selectivity of astemizole.

The favorable effects of the antihistamine astemizole seen in several translational PD studies might be due to mechanisms other than the inhibition of amyloid aggregation. Previous translational studies have indicated that the symptomatic attenuation of Parkinsonism symptoms by histamine H1-receptor antagonists such as ebastine and levocetirizine may be related to the downregulation of oxidative stress and anti-inflammatory effects [30,31]. A previous in vitro study examined whether astemizole induces autophagy in PK1 neuroblastoma cells and monitored the conversion of the cytosolic protein LC3-I into the autophagosome-bound form LC3-II. Astemizole treatment showed a notable change in the LC3-II/I ratio and led to a doubling of this ratio, suggesting an induction of autophagic function, and thereby aiding in prion clearance. Other several mechanistic studies suggest that astemizole has anti-prion effects by modulating autophagy, including promoting Beclin-1-independent autophagy or altering mammalian target of rapamycin (mTOR) signaling [13,32,33]. Recently, Bamia and coworkers showed that astemizole exerts anti-prion effects in mouse organoid cell culture by inhibiting ribosome-assisted protein folding; it is possible that inhibiting the protein folding activity of the ribosome is a mechanism shared by several anti-prion candidates, including astemizole [34]. Together, these proposed modes of action of astemizole unrelated to the inhibition of amyloid aggregation inhibition can underlie the negative results observed in this study. Astemizole can reduce PrP protein levels on neuronal surfaces by more than half and significantly hinders prion surface replication [12,13]. PrP proteins on the cell surface can bind with Aβ oligomers as receptors and facilitate Aβ synaptotoxicity [12,14]. Drugs such as astemizole, which affect cell surface PrP expression, represent a possible approach for AD in humans. This study has not explored the potential therapeutic effects of astemizole in the context of AD beyond its ability to inhibit Aβ42 aggregation. However, our finding using in situ ThT fluorescence monitoring provides real-time kinetic profiles for the modulating effect of astemizole on Aβ fibrillation. This contributes to our understanding of the potential of such drugs for patients with AD and warrants future investigation.

In summary, we examined if astemizole acted as a general amyloid aggregation inhibitor to block the fibrillation of human proteins prone to misfolding. The effects of astemizole on the PD-associated protein α-syn and the AD-associated protein Aβ42 were monitored in ThT and Congo red spectral assays and TEM studies. This current study found that astemizole blocked the assembly of human Aβ42 protein in vitro but did not affect α-syn aggregation, and these findings neither support nor oppose the potential of astemizole as the therapeutic option for PD. Our results need to be interpreted with caution based on several limits. One such limitation is that this study was based on in vitro experiments and did not use animal models. We also did not utilize Western blot or protein gel analysis to quantitatively assess the effects of astemizole on inhibiting α-syn aggregation in both soluble and insoluble protein fractions. This approach could have provided a more precise measurement of the aggregation process, which is a recognized drawback in this study. Other limitations include the lack of other forms of α-syn, such as the oligomeric or the mutated forms (i.e., A53T) of synuclein, which can influence the generalizability of these findings. Considering the known safety issues associated with astemizole, however, a detailed understanding of the drug’s mechanism of action is needed before astemizole can be studied in patients with PD.

## Data Availability

The datasets generated during and/or analyzed during this study are available from the corresponding author on request.

## References

[1] Scheckel C., Aguzzi A. (2018). Prions, prionoids and protein misfolding disorders. Nat. Rev. Genet..

[2] Steiner J.A., Quansah E., Brundin P. (2018). The concept of alpha-synuclein as a prion-like protein: Ten years after. Cell Tissue Res..

[3] Walsh D.M., Selkoe D.J. (2016). A critical appraisal of the pathogenic protein spread hypothesis of neurodegeneration. Nat. Rev. Neurosci..

[4] Baerends E., Soud K., Folke J., Pedersen A.K., Henmar S., Konrad L., Lycas M.D., Mori Y., Pakkenberg B., Woldbye D.P.D. (2022). Modeling the early stages of Alzheimer’s disease by administering intracerebroventricular injections of human native Aβ oligomers to rats. Acta Neuropathol. Commun..

[5] Ruiz-Riquelme A., Lau H.H.C., Stuart E., Goczi A.N., Wang Z., Schmitt-Ulms G., Watts J.C. (2018). Prion-like propagation of β-amyloid aggregates in the absence of APP overexpression. Acta Neuropathol. Commun..

[6] Olsson T.T., Klementieva O., Gouras G.K. (2018). Prion-like seeding and nucleation of intracellular amyloid-β. Neurobiol. Dis..

[7] Henrich M.T., Geibl F.F., Lakshminarasimhan H., Stegmann A., Giasson B.I., Mao X., Dawson V.L., Dawson T.M., Oertel W.H., Surmeier D.J. (2020). Determinants of seeding and spreading of α-synuclein pathology in the brain. Sci. Adv..

[8] Van Den Berge N., Ulusoy A. (2022). Animal models of brain-first and body-first Parkinson’s disease. Neurobiol. Dis..

[9] Aguzzi A., Lakkaraju A.K.K. (2016). Cell Biology of Prions and Prionoids: A Status Report. Trends Cell Biol..

[10] Chong C.R., Chen X., Shi L., Liu J.O., Sullivan D.J. (2006). A clinical drug library screen identifies astemizole as an antimalarial agent. Nat. Chem. Biol..

[11] Di L., Kerns E.H., Fan K., McConnell O.J., Carter G.T. (2003). High throughput artificial membrane permeability assay for blood-brain barrier. Eur. J. Med. Chem..

[12] Karapetyan Y.E., Sferrazza G.F., Zhou M., Ottenberg G., Spicer T., Chase P., Fallahi M., Hodder P., Weissmann C., Lasmézas C.I. (2013). Unique drug screening approach for prion diseases identifies tacrolimus and astemizole as antiprion agents. Proc. Natl. Acad. Sci. USA.

[13] Kocisko D.A., Baron G.S., Rubenstein R., Chen J., Kuizon S., Caughey B. (2003). New Inhibitors of Scrapie-Associated Prion Protein Formation in a Library of 2,000 Drugs and Natural Products. J. Virol..

[14] Aguzzi A., Lakkaraju A.K.K., Frontzek K. (2018). Toward Therapy of Human Prion Diseases. Annu. Rev. Pharmacol. Toxicol..

[15] Rojo L.E., Alzate-Morales J., Saavedra I.N., Davies P., Maccioni R.B. (2010). Selective interaction of lansoprazole and astemizole with tau polymers: Potential new clinical use in diagnosis of Alzheimer’s disease. J. Alzheimers Dis..

[16] Sun A.G., Lin A.Q., Huang S.Y., Huo D., Cong C.H. (2016). Identification of potential drugs for Parkinson’s disease based on a sub-pathway method. Int. J. Neurosci..

[17] Styczyńska-Soczka K., Zechini L., Zografos L. (2017). Validating the Predicted Effect of Astemizole and Ketoconazole Using a Drosophila Model of Parkinson’s Disease. Assay Drug Dev. Technol..

[18] Tahaei Gilan S.S., Yahya Rayat D., Mustafa T.A., Aziz F.M., Shahpasand K., Akhtari K., Salihi A., Abou-Zied O.K., Falahati M. (2019). α-synuclein interaction with zero-valent iron nanoparticles accelerates structural rearrangement into amyloid-susceptible structure with increased cytotoxic tendency. Int. J. Nanomed..

[19] Narkiewicz J., Giachin G., Legname G. (2014). In vitro aggregation assays for the characterization of α-synuclein prion-like properties. Prion.

[20] Gadhave K., Bhardwaj T., Uversky V.N., Vendruscolo M., Giri R. (2021). The signal peptide of the amyloid precursor protein forms amyloid-like aggregates and enhances Aβ42 aggregation. Cell Rep. Phys. Sci..

[21] Sanz F.J., Solana-Manrique C., Muñoz-Soriano V., Calap-Quintana P., Moltó M.D., Paricio N. (2017). Identification of potential therapeutic compounds for Parkinson’s disease using Drosophila and human cell models. Free Radic. Biol. Med..

[22] Hernández-Parra H., Cortés H., Avalos-Fuentes J.A., Del Prado-Audelo M., Florán B., Leyva-Gómez G., Sharifi-Rad J., Cho W.C. (2022). Repositioning of drugs for Parkinson’s disease and pharmaceutical nanotechnology tools for their optimization. J. Nanobiotechnology.

[23] Tian J., Vandermosten L., Peigneur S., Moreels L., Rozenski J., Tytgat J., Herdewijn P., Van den Steen P.E., De Jonghe S. (2017). Astemizole analogues with reduced hERG inhibition as potent antimalarial compounds. Bioorg. Med. Chem..

[24] Olasińska-Wiśniewska A., Olasiński J., Grajek S. (2014). Cardiovascular safety of antihistamines. Postep. Dermatol. Alergol..

[25] Lindquist M., Edwards I.R. (1997). Risks of non-sedating antihistamines. Lancet.

[26] Rajan R., Ahmed S., Sharma N., Kumar N., Debas A., Matsumura K. (2021). Review of the current state of protein aggregation inhibition from a materials chemistry perspective: Special focus on polymeric materials. Mater. Adv..

[27] Almeida Z.L., Brito R.M.M. (2022). Amyloid Disassembly: What Can We Learn from Chaperones?. Biomedicines.

[28] Wentink A., Nussbaum-Krammer C., Bukau B. (2019). Modulation of Amyloid States by Molecular Chaperones. Cold Spring Harb. Perspect. Biol..

[29] Webster J.M., Darling A.L., Uversky V.N., Blair L.J. (2019). Small Heat Shock Proteins, Big Impact on Protein Aggregation in Neurodegenerative Disease. Front. Pharmacol..

[30] Ayaz M., Anwar F., Saleem U., Shahzadi I., Ahmad B., Mir A., Ismail T. (2022). Parkinsonism Attenuation by Antihistamines via Downregulating the Oxidative Stress, Histamine, and Inflammation. ACS Omega.

[31] Nuutinen S., Panula P. (2010). Histamine in neurotransmission and brain diseases. Adv. Exp. Med. Biol..

[32] Nelson M.P., Shacka J.J. (2013). Autophagy Modulation in Disease Therapy: Where Do We Stand?. Curr. Pathobiol. Rep..

[33] López-Pérez Ó., Badiola J.J., Bolea R., Ferrer I., Llorens F., Martín-Burriel I. (2020). An Update on Autophagy in Prion Diseases. Front. Bioeng. Biotechnol..

[34] Bamia A., Sinane M., Naït-Saïdi R., Dhiab J., Keruzoré M., Nguyen P.H., Bertho A., Soubigou F., Halliez S., Blondel M. (2021). Anti-prion Drugs Targeting the Protein Folding Activity of the Ribosome Reduce PABPN1 Aggregation. Neurotherapeutics.

